# Association of *BLK* and *BANK1* Polymorphisms and Interactions With Rheumatoid Arthritis in a Latin-American Population

**DOI:** 10.3389/fgene.2020.00058

**Published:** 2020-02-20

**Authors:** Julian Ramírez-Bello, José M. Fragoso, Isidro Alemán-Ávila, Silvia Jiménez-Morales, Alma D. Campos-Parra, Rosa Elda Barbosa-Cobos, José Moreno

**Affiliations:** ^1^ Unidad de Investigación, Hospital Juárez de México, Mexico City, Mexico; ^2^ Laboratorio de Biología Molecular, Instituto Nacional de Cardiología, Mexico City, Mexico; ^3^ Laboratorio de Genómica del Cáncer, Instituto Nacional de Medicina Genómica, Mexico City, Mexico; ^4^ Laboratorio de Genómica, Instituto Nacional de Cancerología, Mexico City, Mexico; ^5^ Servicio de Reumatología, Hospital Juárez de México, Mexico City, Mexico; ^6^ Dirección de Investigación, Hospital Juárez de México, Mexico City, Mexico

**Keywords:** rheumatoid arthritis, single nucleotide variants, gene interaction, susceptibility, association

## Abstract

**Introduction:**

*BLK* has been identified as a risk factor to rheumatoid arthritis (RA) primarily in Asian or European-derived populations. However, this finding has not been evaluated in other populations such as Latin-Americans, except for Colombians. On the other hand, *BANK1* single nucleotide variants (SNVs) have been scarcely studied in RA patients.

**Objective:**

The aim of this study was to determine whether the BLK rs2736340T/C, rs13277113A/G, and BANK1 rs10516487G/A (R61H) and rs3733197G/A (A383T) polymorphisms are risk factors to RA in a sample of patients from Central Mexico.

**Materials and Methods:**

We studied 957 women; 487 controls and 470 patients with RA by means of a TaqMan® SNP genotyping assay with fluorescent probes for the *BLK* rs13277113A/G, rs2736340T/C and *BANK1* 10516487G/A (R61H) and rs3733197G/A (A383T) variants.

**Result:**

The *BLK* rs2736340T/C and rs13277113A/G variants were associated with risk for RA: C vs T; OR 1.39, *p* = 0.001, and G vs A; OR 1.37, *p* = 0.004, respectively. In addition, there was also an association between *BANK1* R61H and RA: A vs G; OR 1.49, *p* = 0.003, but no with *BANK1* A383T. We also identified an interaction significant between genotypes of *BLK* rs2736340T/C-*BANK1* rs10516487G/A and RA: OR 1.65, *p* = 0.0001.

**Conclusions:**

Our data suggest that both *BLK* and *BANK1* confer susceptibility to RA in Mexican patients. The individual association of *BANK1* rs1054857G/A with RA had not been previously reported in a particular population (except for pooled patients from several countries), therefore, our study presents the first evidence of association between this *BANK1* variant and RA.

## Introduction

Genetic association to autoimmune diseases (ADs) includes variants of genes coding for proteins involved in B lymphocyte antigen receptor signaling, of which *BLK* and *BANK1* are components of the B-cell signalosome (Suthers and Sarantopoulos, 2017). The *BLK* protein is a src family non-receptor tyrosine kinase mainly expressed by B-cells, where besides B-cell receptor signaling, it plays a role in development (Akerblad and Sigvardsson, 1999; Tretter et al., 2003), meanwhile, the *BANK1* protein is an adaptor/scaffold primarily expressed in B-lymphocytes, which plays an important role in activation and signaling (Kozyrev et al., 2008; Castillejo-Lopez et al., 2012). It has been shown that *BLK*, similar to other members of the *src* family interacts with *BANK1* (Castillejo-Lopez et al., 2012). Thus, *BLK* and *BANK1* proteins have an important role in both B-cell signaling and activation.

In 2009 and 2011, two genome wide association studies (GWAS) carried out in European and Asian derived-populations, respectively, identified some single nucleotide variants (SNVs) in the *BLK* gene to be associated with risk for rheumatoid arthritis (RA) (Gregersen et al., 2009; Freudenberg et al., 2011). However, other GWA or candidate gene studies have failed to replicate these findings (Génin et al., 2013; Jiang et al., 2014; Orozco et al., 2014; Bossini-Castillo et al., 2015; Zhu et al., 2016; Julià et al., 2016; Saxena et al., 2017), except in patients from United Kingdom, Colombia, and China (Deshmukh et al., 2011; Orozco et al., 2011; Viatte et al., 2012; Huang et al., 2017). On the other hand, the *BANK1* gene, which was identified as an important risk factor for systemic lupus erythematosus (SLE) (Kozyrev et al., 2008), does not appear to be associated with RA through GWA (Gregersen et al., 2009; Freudenberg et al., 2011; Jiang et al., 2014; Orozco et al., 2014; Bossini-Castillo et al., 2015; Julià et al., 2016; Saxena et al., 2017) or candidate gene studies (Suarez-Gestal et al., 2009; Orozco et al., 2011; Génin et al., 2013; Huang et al., 2017), except for the *BANK1* rs3733197G/A (Ala383Thr; non-synonymous polymorphism) SNV, which showed an association with RA in Spanish and Argentine patients (Orozco et al., 2009).

Although different *BLK* and *BANK1* SNVs have been examined in patients with RA, the results are uncertain because some reports show an association with this AD (Gregersen et al., 2009; Orozco et al., 2009; Deshmukh et al., 2011; Freudenberg et al., 2011; Orozco et al., 2011; Viatte et al., 2012; Huang et al., 2017), while others have not replicated this finding (Suarez-Gestal et al., 2009; Génin et al., 2013; Jiang et al., 2014; Orozco et al., 2014; Bossini-Castillo et al., 2015; Julià et al., 2016; Zhu et al., 2016; Saxena et al., 2017). In addition, *BLK* (except in patients from Colombia) and *BANK1* (except in one study which evaluated patients from Argentina and Mexico) variants have been scarcely explored in different Latin-American populations with RA (Orozco et al., 2009; Deshmukh et al., 2011). Therefore, our aim was to examine the possible associations of *BLK* rs13277113A/G and rs2736340T/C and *BANK1* rs10516487C/T (R61H) and rs3733197G/A (A383T) SNVs with RA in Mexican patients, in addition to the interactions between *BLK* and *BANK1* genotypes and this AD.

## Material and Methods

### Patients and Controls

The present study included 957 individuals from Central Mexico (from Mexico City, States of Mexico, Morelos, Hidalgo, and Puebla), 470 with RA and 487 controls. Because our sample was formed by a proportion of women/men affected with RA of 93.6% and 6.3% (data not shown), respectively, and to avoid any possible bias as a result of the low number of males with possibly distinct features, we decided to exclude men from our study. Previous studies in Mexico have shown a similar proportion of women/men affected with RA (Muñoz-Valle et al., 2017; Zaragoza-García et al., 2019), which is very different from what was reported in Caucasians, where RA is three times more frequent in women than in men (Scott et al., 2010). Therefore, all cases and controls were unrelated women >18 years old. Healthy individuals included in this study were obtained from the blood bank laboratory of Hospital Juárez de México (HJM) and had no family history of ADs or inflammatory diseases, and no personal history of obesity, hypertension, cancer, and allergy. All RA patients fulfilled the 2010 ACR-EULAR criteria (Aletaha et al., 2010) and were recruited from the Department of Rheumatology, HJM. Patients and controls were matched by ancestry and gender. Studies were conducted in compliance with the Declaration of Helsinki. Additionally, all individuals included in our study signed a written informed consent. This protocol was approved by the Ethics and Research Committees of the HJM (registry number 0446/18-I).

### DNA Extraction

Peripheral blood samples (5–8 ml of EDTA-treated) were used to isolate genomic DNA from leukocytes by means of the Invisorb Bood Universal Kit (Stratec Molecular GmbH, Berlin, Germany), according to manufacturer's specifications. DNA samples were quantified, diluted (5 ng/µl), and stored at −20°C until needed.

### Genotyping

We used a TaqMan^®^ SNP genotyping assay (Applied Biosystems, Foster City, CA) for the *BLK* rs13277113A/G and rs2736340T/C and *BANK1* rs10516487C/T (R61H) and rs3733197G/A (A383T) genotypes. The Bio-Rad CFX Manager 3.1 software implemented in the CFX96 Touch TM Real-Time PCR of Bio-Rad (Bio-Rad, California, USA) was used to determinate the *BLK* and *BANK1* genotypes in an allelic discrimination plot. The distribution of each *BLK* and *BANK1* genotype was determined by two independent researchers. Additionally, 70% of all samples (including cases and controls) were genotyped twice with a reproducibility of 100%. PCR conditions for each amplicon and sample were as follows: 10 ng DNA per sample, 2.5 μl of TaqMan^®^ Universal Master Mix (2X) (Applied Biosystems, Foster City, CA), 2.435 μl of nuclease-free water, and 0.065 μl of TaqMan probes (Applied Biosystems). The PCR protocol for amplification was as follows: pre-PCR (one cycle); at 50°C for 2 min and at 95°C for 8 min, followed by 45 cycles of denaturing at 95°C for 15 s and annealing an extension at 60°C for 1 min.

### Statistical Analysis

We used the Finetti software to evaluate the Hardy-Weinberg equilibrium (HWE) for all *BLK* and *BANK1* SNVs (a *p*-value < 0.05 in both cases and controls was indicative of deviation from HWE) (https://ihg.gsf.de/cgi-bin/hw/hwa1.pl). We used the Epidat program (http://www.sergas.es/MostrarContidos_N3_T01.aspx?IdPaxina=62715) to examine the genetic association between *BLK* and *BANK1* polymorphisms and RA under the allelic, codominant, dominant, and recessive genetic models. All results were corrected through the Bonferroni correction test (0.05/4 SNVs; *p =* 0.0125), with a *p*-value between 0.05 and 0.0125 considered as nominal significance, and *p*-value ≤0.0125 as significant. Moreover, all *p*-values obtained of different inheritance genetic models were evaluated by logistic regression and adjusted for age and city of origin. The Haploview program was used to obtain the haplotypes and the linkage disequilibrium (LD) among *BLK* and *BANK1* markers (Barrett et al., 2005). Interactions between genotypes of high and low risk of the four *BLK* and *BANK1* variants for both cases and controls were obtained by the multifactor dimensionality reduction (MDR) program, which allows the evaluation of the quality of gene-gene interactions to be measured by means of two-way contingency tables (Moore et al., 2006). Quanto software (http://hydra.usc.edu/gxe) was used to determine the statistical power of our study. We took into account the minor allele frequency (MAF) of the four *BLK* and *BANK1* SNVs in controls, in addition to a recessive genetic model, the proportion of cases-controls, an odd ratio (OR) of 2.0, the prevalence of RA in Mexicans (Peláez-Ballestas et al., 2011) as well as the sample size.

## Results

### HWE and Statistical Power in our Study Population

The *BLK* and *BANK1* genotypes were in HWE both in patients with RA and controls, except for *BANK1* rs3733197G/A in controls, which had a weak deviation from HWE (*p =* 0.02). We identified a statistical power >99% in our study taking into account the MAF of the four *BLK* and *BANK1* variants in controls (data not shown).

### Allele and Genotype Frequencies of the *BLK* Polymorphisms and Association Analysis

*BLK* rs13277113A and rs2736340T major alleles were associated with RA (G vs A; OR 1.37, *p =* 0.002, and C vs T; OR 1.39, *p =* 0.001, respectively [**Table 1**]). We also identified an association of *BLK* rs13277113A/G with RA under the recessive (OR 1.52, *p* = 0.0013), and codominant (OR 1.52, *p =* 0.0055) models but not under the dominant model (**Table 1**). Because the *BLK* rs13277113A/G variant is in high LD with rs2736340T/C (r^2^ ≈ 1), we also observed a similar association between *BLK* rs2736340T/C and RA, C v T; OR 1.39, *p =* 0.001, and under the recessive and codominant models, the OR were 1.55, (*p =* 0.0008) and 2.58 (*p =* 0.0036), respectively (**Table 1**).

**Table 1:** Genotypic and allelic frequencies of the *BLK* rs13277113A/G and rs2736340T/C SNVs and association analysis in RA patients and healthy individuals.

*Gene* *SNV*	Model	Genotype	*RA*	*Controls*	*OR*	p*
			n (%)	n (%)	95% CI	
*BLK*	Codominant	AA	268 (57.0)	226 (46.4)	1.52 (0.95–2.45)	0.0055
*rs13277113A/G*		AG	167 (35.5)	215 (44.1)	1.00 (0.62–1.63)	NS
		GG	35 (7.5)	46 (9.4)		
	Alleles	A	703 (74.8)	667 (68.5)	1.37 (1.12–1.67)	0.002
		G	237 (25.2)	307 (31.5)		
	Dominant	AA+AG	435 (92.5)	441 (90.5)	1.27 (0.80–2.01)	NS
		GG	35 (7.5)	46 (9.4)		
	Recessive	AA	268 (57.0)	226 (46.4)	1.52 (1.18–1.96)	0.0013
		AG+GG	202 (43.0)	261 (53.6)		
*BLK*	Codominant	TT	269 (57.2)	225 (46.2)	1.58 (1.00–2.56)	0.0036
*rs2736340T/C*		TC	167 (35.5)	216 (44.4)	1.03 (0.63–1.68)	NS
		CC	34 (7.2)	46 (9.4)		
	Alleles	T	705 (75.0)	666 (68.4)	1.39 (1.14–1.70)	0.001
		C	235 (25.0)	308 (31.6)		
	Dominant	TT+TC	436 (92.8)	441 (90.5)	1.31 (0.82–2.08)	NS
		CC	34 (7.2)	46 (9.4)		
	Recessive	TT	269 (57.2)	225 (46.2)	1.55 (1.20–2.00)	0.0008
		TC+CC	201 (42.8)	262 (53.8)		

SNV, single nucleotide variant; RA, rheumatoid arthritis; OR, odds ratio; CI, confidence interval; RA, rheumatoid arthritis. *p < 0.05, statistically significant. The inheritance genetic models were calculated by logistic regression and adjusted for age and city. NS, not significant.

### Allele and Genotype Frequencies of the *BANK1* Polymorphisms and Association Analysis

Our data shows that the *BANK1* rs10516487G/A variant is a risk factor to RA. Of note, we identified *BANK1* rs10516487A as the major allele associated with susceptibility to this AD: A vs G; OR 1.49, *p =* 0.003 (**Table 2**). We also observed an association under the recessive (OR 1.47, *p =* 0.01) and codominant (OR 2.16, *p =* 0.012) models and a nominal significance under the dominant model (OR 2.78, *p =* 0.036) (**Table 2**). No association was identified between *BANK1* rs3733197G/A and RA under any genetic model (**Table 2**).

**Table 2:** Genotypic and allelic frequencies of the *BANK1* rs10516487G/A and rs3733197G/A SNVs and association analysis in RA patients and healthy individuals.

*Gene* *SNV*	Model	Genotype	*RA*	*Controls*	*OR*	P*
			n (%)	n (%)	95% CI	
*BANK1*	Codominant	GG	369 (78.5)	347 (71.2)	3.00 (1.08–8.38)	NS
*rs10516487G/A*		GA	96 (20.4)	125 (25.7)	2.16 (0.76–6.18)	0.012
*R61H*		AA	5 (1.1)	15 (3.1)		
	Alleles	G	834 (88.7)	819 (84.1)	1.49 (1.14–1.94)	0.003
		A	106 (11.3)	155 (15.9)		
	Dominant	GG+GA	465 (98.9)	472 (96.9)	2.78 (1.00–7.74)	0.036
		AA	5 (1.1)	15 (3.1)		
	Recessive	GG	369 (78.5)	347 (71.2)	1.47 (1.10–1.98)	0.01
		GA+AA	101 (21.5)	140 (28.8)		
*BANK1*	Codominant	GG	308 (65.5)	307 (63.0)	1.27 (0.74–2.20)	NS
*rs3733197G/A*		GA	137 (29.2)	148 (30.4)	1.18 (0.67–2.10)	NS
*A383T*		AA	25 (5.3)	32 (6.6)		
	Alleles	G	753 (80.1)	762 (78.2)	1.12 (0.90–1.40)	NS
		A	187 (19.9)	212 (21.8)		
	Dominant	GG+GA	445 (94.7)	455 (93.4)	1.24 (0.72–2.14)	NS
		AA	25 (5.3)	32 (6.6)		
	Recessive	GG	308 (65.5)	307 (63.0)	1.11 (0.85–1.45)	NS
		GA+AA	162 (34.5)	180 (37.0)		

SNVs, single nucleotide variants; RA, rheumatoid arthritis; OR: odds ratio; CI, confidence interval; RA, rheumatoid arthritis. *p < 0.05, statistically significant. The inheritance genetic models were calculated by logistic regression and adjusted for age and city. NS, Not significant.

### Haplotypes and LD Analysis

We identified a pair of haplotypes between the two *BLK* SNVs in RA patients because of a strong LD among them (data not shown), the TA haplotype, which carries the major alleles of the *BLK* rs2736340T/C and rs13277113A/G SNVs showed an association with susceptibility to RA; OR 1.36, *p =* 0.0023, *p*c 0.0024 after 100,000 permutations (data not shown). We also identified an association between the AA haplotype carrying the *BANK1* rs10516487G/A and rs3733197G/A minor alleles and protection against RA; OR 0.68, *p* = 0.01 (*p*c = 0.038) (**Table 3**), indicating that the *BANK1* rs10516487G major allele together with the rs3733197A minor allele; GA haplotype, is not associated with RA. Regarding the *BANK1* variants, there was no LD between the two *BANK1* SNVs (data not shown).

**Table 3:** Haplotype frequencies and association analysis between *BANK1* SNVs in RA patients and controls. The order of the *BANK1* SNVs is: rs10516487G/A and rs3733197G/A.

Haplotype	RA(%)	Controls (%)	OR	95% CI	*P*	*Pc*
*BANK1*						
GG	78.2	75.5	1.17	0.94–1.44	NS	NS
AA	9.4	13.1	0.68	0.51–0.91	0.01	0.038
GA	10.5	8.6	1.25	0.92–1.69	NS	NS
AG	1.9	2.8	0.68	0.37–1.25	NS	NS

RA, rheumatoid arthritis; OR, odds ratio; CI, confidence interval p < 0.05, statistically significant. pc: corrected p-value after 100,000 permutations.

### Genetic Interactions Between *BLK* and *BANK1* Genotypes and RA

**Figure 1:**
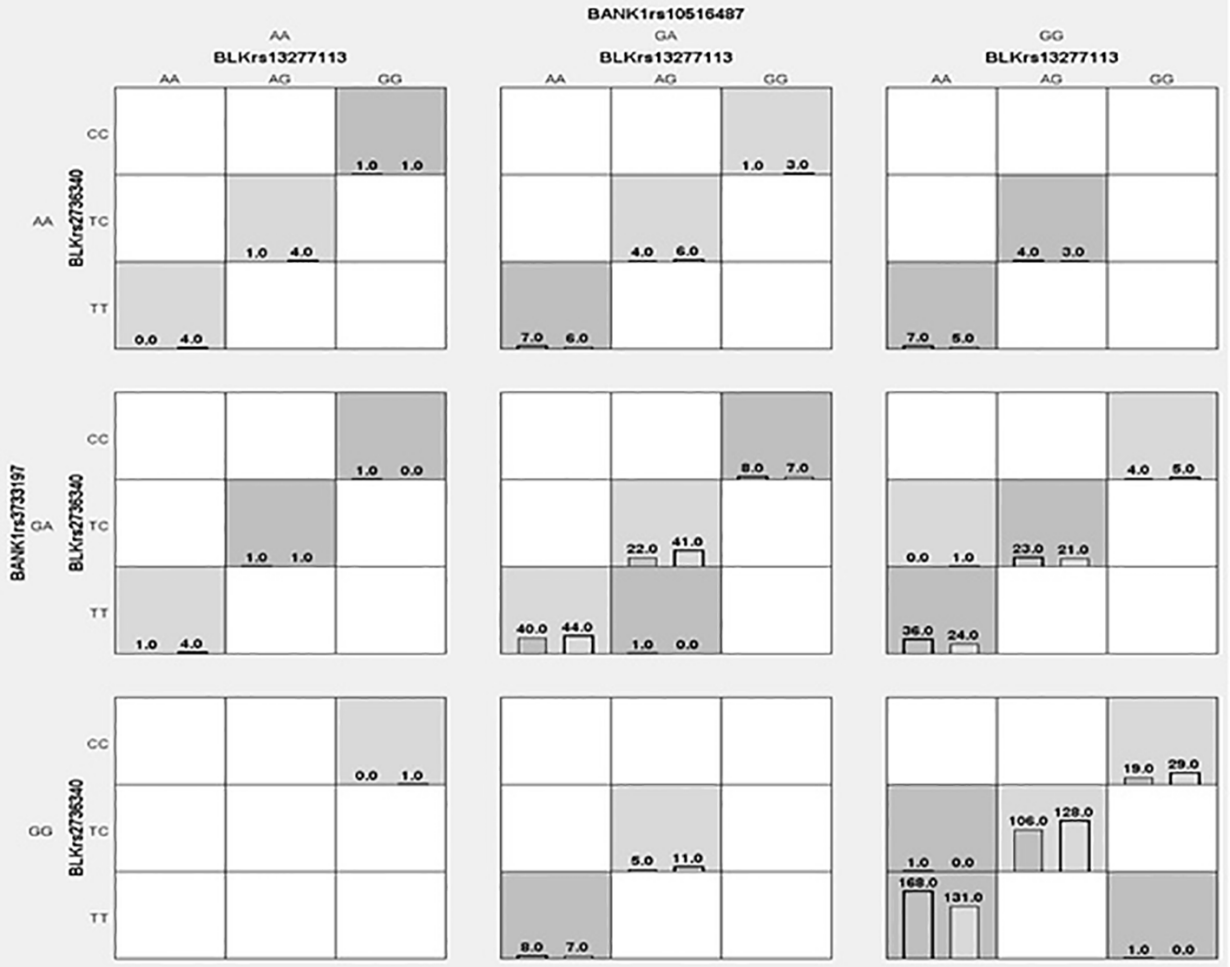
Distribution of genotypes in cases and controls for *BLK* rs13277113A/G-rs2736340T/C and *BANK1* rs10516487G/A-rs3733197G/A. Each cell shows counts of cases (left) and controls (right). Dark-shaded cells represent “high-risk” genotypes, meanwhile, lighter-shaded cells represent “low-risk” genotypes. White-shades cells represent empty, that is, there are no cases-controls with genotypes.

The distribution of interactions between the *BLK* rs2736340T/C-rs13277113A/G and *BANK1* rs10516487G/A-rs3733197G/A genotypes in RA patients and controls are shown in **Figure 1**. We identified three genetic interactions, of which the best model was represented by *BLK* rs2736340T/C and *BANK1* rs10516487G/A (testing accuracy 0.5443, and cross-validation consistency 10/10), this interaction also showed an association with susceptibility to RA (OR 1.65, *p =* 0.0001) (**Table 4**). Of note, both *BLK* rs36340T/C (this variant showed almost a complete LD, r^2^≈1, with *BLK* rs13277113A/G) and *BANK1* rs10516487G/A variants were previously identified to be associated with RA (**Tables 1** and **2**). Because of strong LD between *BLK* rs2736340T/C and rs13277113A/G SNVs, we identified in the dendrogram analysis a redundancy between these markers (data not shown). In addition, we also identified the *BANK1* rs3733197G/A variant with an independent effect (data not shown). The second best interaction model was formed by the *BLK* rs2736340T/C-rs13277113A/G plus *BANK1* rs10516487G/A-rs3733197G/A genotypes (testing accuracy; 0.5381, and cross-validation consistency; 10/10), this interaction was also associated with RA (OR 1.79, *p* < 0.0001). Finally, we did not observe a synergistic interaction between the four *BLK* an *BANK1* variants (data not shown).

**Table 4:** Gene-gene interaction models between *BLK* and *BANK1* SNVs in cases with RA and controls.

Number of factors	Best model*	Training accuracy	Testing accuracy	CVC**	X^2^	*p* value^≠^	OR (CI 95%)
1	*BLK* (rs2736340)	0.5552	0.5552	10/10	11.66	0.0006	1.56 (1.21–2.01)
2	*BLK* (rs2736340)-*BANK1* (rs10516487)	0.5636	0.5434	10/10	14.90	0.0001	1.65 (1.28–2.13)
3	*BLK* (rs2736340)-*BANK1*(rs10516487)-(rs3733197)	0.5713	0.528	6/10	18.43	<0.0001	1.75 (1.36–2.33)
4	*BLK* (rs2736340)-(rs13277113)-*BANK1*(rs10516487)-(rs3733197)	0.5739	0.5381	10/10	20.14	<0.0001	1.79 (1.39–2.32)

*The best model is referred to as the one with the maximum testing accuracy and maximum CVC.

**Cross-validation consistency.

^≠^p-values obtained from the statistics whole dataset.

## Discussion

The study of gene polymorphism associations to diseases in different ethnic groups is an important means to close down on the likelihood that particular genes and specific variants to the disease. Here we studied two SNVs of the *BLK* gene, and two of *BANK1* (both of them part of the B cell signalosome) for their association to RA in Mexican patients. Association of *BLK* SNVs to RA was initially documented in North American and Korean patients (Gregersen et al., 2009; Freudenberg et al., 2011), and later in British, Colombian, and Han Chinese patients (Deshmukh et al., 2011; Orozco et al., 2011; Viatte et al., 2012; Huang et al., 2017). Nevertheless, other GWA or candidate gene studies carried out in some Arab countries and Europeans from the Netherlands and Spain, and even another Chinese group failed to replicate this finding (Suarez-Gestal et al., 2009; Génin et al., 2013; Jiang et al., 2014; Bossini-Castillo et al., 2015; Julià et al., 2016; Zhu et al., 2016; Saxena et al., 2017). As far as we know, the only study conducted in Latin-Americans thus far, found an association of both *BLK* rs13277113A/G and rs2736340T/C SNVs and risk for RA in Colombians (Deshmukh et al., 2011). Our current findings confirm such association in Mexican RA patients. On the other hand, in European-derived populations, *BLK* association to RA has only been identified in patients from the UK (Orozco et al., 2011; Viatte et al., 2012). It is important to note that in Chinese population, one candidate gene study found an association between *BLK* rs13277113A/G and RA (Huang et al., 2017), which was not previously identified in a GWAS (Jiang et al., 2014). GWAS allow the identification of many genes associated with any disease, including RA or other ADs (Gregersen et al., 2009; Freudenberg et al., 2011; Kozyrev et al., 2008; Rodríguez-Elías et al., 2016). However, in these studies, several hundreds or thousands of SNVs are removed, particularly because of their high error rates, such as those with an excess missing genotype (Anderson et al., 2010). Possible explanations for this are: a) the probes for the genotyping of *BLK* rs13277113A/G and rs2736340T/C (or SNVs in high LD for these variants) SNVs might have been lost in that process, b) the controls had a deviation from the HWE, c) these variants had no associations at genome-wide significance level (p < 5×10^−8^), among others. All these caveats must be considered to explain the controversial results found among the aforementioned studies. To solve this, it would be useful to carry out replications with candidate gene studies in the different populations to confirm or rule out these findings. It is important to note that in controls from North America, Spain (or European-derived populations), Colombia, and Africa, *BLK* rs2736340T or the *BLK* rs13277113A are the minor alleles (Hom et al., 2008; Gregersen et al., 2009; Orozco et al., 2009; Deshmukh et al., 2011; Sánchez et al., 2011; Génin et al., 2013; Guthridge et al., 2014; Martínez-Bueno et al., 2018) whereas in our population these were the major alleles as reported in the 1000 Genomes Project for Mexicans (Mexican-Americans from Los Angeles, CA) (https://www.ncbi.nlm.nih.gov/variation/tools/1000genomes/) as well as in other Asian populations like Japanese and Chinese (Génin et al., 2013; Dang et al., 2016; Huang et al., 2017) (**Table 5**). We conclude, hence, that in our population the *BLK* rs2736340T and/or rs13277113A major alleles are associated to risk for RA.

**Table 5:** Frequency of the *BLK* rs13277113G, rs2736340T and *BANK1* rs10516487A-rs3733197A alleles associated with RA susceptibility in our study, in Mexican-Americans who lives in the Ángeles as well as in European, Asian, and African (or African-American)-derived populations. All data presented here are from control individuals.

	Our study (%)	Mexican-American (1000 Genome Project) (%)	Caucasian (%)	Ref.	Asian (%)	Ref.	African or African-American(%)	Ref.
***BLK***								
rs13277113A/G								
A allele	68.5	64.1	22.9	(Hom et al., 2008)	70.2	(Huang et al., 2017)	13.1	(Sánchez et al., 2011)
rs2736340C/T								
T allele	68.4	64.1	23.2	(Hom et al., 2008)	70.9	(Dang et al., 2016)	13.4	(Guthridge et al., 2014)
***BANK1***								
rs10516487G/A								
A allele	15.9	14.8	27.6	(Orozco el at., 2009)	13.9	(Dang et al., 2016)	26.0	(Martínez-Bueno et al., 2018)
rs3733197G/A								
A allele	21.8	20.3	30.4	(Orozco et al., 2009)	22.0	(Huang et al., 2017)	21.5	(Bueno et al., 2018)

Functionally, the rs13277113A allele has been associated with lower levels of *BLK* mRNA in transformed B-cell lines (Hom et al., 2008). However, it is possible that this *BLK* SNV is in LD with another *BLK* variant truly associated with risk for RA. In this manner, *BLK* rs13277113A appears to belong to a haplotype associated with RA, such as the *BLK* rs922483 variant (which is in high LD; r^2^ > 0.7 with both *BLK* rs13277113 and rs2736340T/C), which affects the *BLK* mRNA and protein expression early in B cell ontogeny (Simpfendorfer et al., 2012). Moreover, another study found that the rs922483 and rs1382568 variants located within the proximal *BLK* promoter and in the upstream alternative *BLK* promoter, respectively (both BLK variants are in high LD with rs13277113; r^2^ > 0.74 in Europeans), affect the promoter activity in B progenitor cell lines (Guthridge et al., 2014).

On the other hand, the two *BANK1* rs10516487C/T (R61H) and rs3733197G/A (A383T) variants have been scarcely studied in patients with RA (Orozco et al., 2009; Suarez-Gestal et al., 2009; Orozco et al., 2011; Génin et al., 2013; Huang et al., 2017), having found only one association with *BANK1* rs3733197G/A in RA patients from Spain and Argentina (Orozco et al., 2009). In that same study, a sample of Mexican RA patients was examined for *BANK1* rs10516487C/T, rs3733197G/A, and rs17266594T/C showing no association. There was, however, an association of RA with *BANK1* rs10516487G/A and rs3733197G/A when the cases and controls of all the countries were pooled. Our current results identified an individual association between *BANK1* rs10516487G/A and RA in Mexican patients, which is at a variance with what was published by Orozco et al. (2009). A possible explanation for this discrepancy could be the different sample sizes between both studies. Our study included 470 RA patients and 487 controls, whereas they studied 278 patients and 272 controls. In addition, our cases and controls are from Central Mexico, whereas Orozco et al. did not reveal the place of origin of their cases and controls (Orozco et al., 2009). The Mexican Republic is a large country with a great population admixture of several ethnic origins, which are not evenly distributed, e.g. Mexico City, located in central Mexico, is formed by approximately 50% Amerindian, 45% Caucasian, and 5% African ancestry (Martinez-Marignac et al., 2007). On the other hand, regions located in Western or Southeastern Mexico have a markedly different ethnic composition (Martínez-Cortés et al., 2012; Rincón et al., 2016). As far as we can tell, this is the first study showing an individual association between *BANK1* rs1051648T/C and RA. Although some authors have not found association between different *BANK1* or *BLK* alleles and risk for RA, in Spanish and Chinese patients an association of an interaction of certain *BANK1*-*BLK* genotype with RA has been reported (Génin et al., 2013; Huang et al., 2017). Moreover, is has been documented a physical and genetic interaction between *BLK* and *BANK1* (Castillejo-Lopez et al., 2012), which would support our finding of a *BLK-BANK1* genetic interaction associated to RA (Castillejo-Lopez et al., 2012; Génin et al., 2013; Huang et al., 2017). According to our results, the best model of interaction was *BLK* rs2736340T/C-*BANK1* rs10516487G/A.

Among the four studied SNPs, it has been reported that rs13277113A/G and rs2736340T/C could have a potential eQTL effect on *FAM167A* and *BLK* genes in monocytes and lymphoblastic cell lines suggesting that these variants are involved in the physiopathology of RA and SLE (Deng et al., 2013a; Deng et al., 2013b). In addition, because both *BLK* variants studied here map near the promoter, they were examined *in silico* by means of expression quantitative trait locus (eQTL) studies (Schmiedel et al., 2018), which point out to a possible overrepresentation of T helper cells, predominantly Th17 and almost any other T helpers, and for the *BLK-FAM167A* locus for naïve B cells (for rs13277113A/G). Although for rs2736340T/C the eQTL associations are similar, there is a slight difference for the *BLK* only on that T cell overrepresentation is Th17 followed by T follicular helper cells. Thus, we can conclude that these associations clearly point to a role of an altered adaptive immunity conferring risk to develop RA. For the *BANK1* variants we did not do an eQTL analysis, because both of them affect the ORF and not the regulatory regions.

A study about the functional consequence of the non-synonymous *BANK1* rs10516487G/A variant (located in exon 2 with a change of arginine to histidine at amino acid position 61 [R61H] of *BANK1*) reported that the product of the *BANK1* rs10516487A allele lacks a binding site for the SRp40 splicing enhancer protein, thereby affecting the splicing efficiency of *BANK1* isoforms (Kozyrev et al., 2012). In addition, the *BANK1* rs10516487GG genotype produces more *BANK1* mRNA and protein than the rs10516487AA genotype. Moreover, the full-length isoform transcript BANK1-R61 generates a larger protein complex and self-association and multimerization than BANK1-H61 (Kozyrev et al., 2012). Another study showed the functional effect of the SLE-associated TGG risk haplotype which is formed by *BANK1* rs17266594T/C, rs10516487G/A, and rs3733197G/A. The first variant is located in a putative branch point of intron 1, the second and third variants (R61H and A383T) are located in exon 2 and 7, respectively. Thus, the TGG risk haplotype is formed by T allele of the rs17266594T/C variant and by Arg61 and Ala383. That study showed that this risk haplotype leads to a decrease of B cell receptor signaling in Ramos B cells and in peripheral B cells. Finally, it was also described in *BANK1* risk carriers that this haplotype appears to result on an increased number of memory B cells (Dam et al., 2016).

Of note, the lack of clinical and laboratory information (bone erosions, rheumatoid nodules, autoantibodies such as anti-CCPs, rheumatoid factor) as well as the absence of mRNA or protein expression studies to correlate with the four *BLK* and *BANK1* variants studied, are some limitations of our study. Thus, at present, it is not possible to determine if these variants are associated with particular traits of RA patients.

In summary, our data indicates that both *BLK* as *BANK1* SNVs studied herein are associated with risk to RA in Mexican patients. On the other hand, the individual association of *BANK1* rs1054857G/A with RA found in the present study had not been identified in a particular population, although one study reported association between this variant and RA only in pooled patients from several countries. The main limitations in our study is the lack of clinical traits and serological markers in or RA patients, as well as the absence of ancestry informative-markers.

## Data Availability Statement

The data has been uploaded to EVA repository Project: PRJEB36187 Analyses: ERZ1284135 www.ebi.ac.uk/eva/?eva-study=PRJEB36187.

## Ethics Statement

This study was conducted in compliance with the Declaration of Helsinki. All individuals included in our study signed a written informed consent. This protocol was approved by the Ethics and Research Committees of the HJM (registry number 0446/18-I). The patients/participants provided their written informed consent to participate in this study.

## Author Contributions

JR-B contributed to the study design, acquired the funding, data analyzed, and wrote the article. IA-Á performed the methodology and contributed with the experiments. JF and AC-P performed the data analysis. JM contributed to the data analysis and wrote the article. SJ-M contributed to the data analysis. RB-C contributed in the explanation of the project to the patients and in the collection of samples.

## Conflict of Interest

The authors declare that the research was conducted in the absence of any commercial or financial relationships that could be construed as a potential conflict of interest.
